# Findings in young adults at colonoscopy from a hospital service database audit

**DOI:** 10.1186/s12876-017-0612-y

**Published:** 2017-04-19

**Authors:** Stephanie Wong, Ilmars Lidums, Christophe Rosty, Andrew Ruszkiewicz, Susan Parry, Aung Ko Win, Yoko Tomita, Sina Vatandoust, Amanda Townsend, Dainik Patel, Jennifer E. Hardingham, David Roder, Eric Smith, Paul Drew, Julie Marker, Wendy Uylaki, Peter Hewett, Daniel L. Worthley, Erin Symonds, Graeme P. Young, Timothy J. Price, Joanne P. Young

**Affiliations:** 1 Department of Gastroenterology, The Queen Elizabeth Hospital, Woodville South 5011, Adelaide, South Australia Australia; 2 Envoi Specialist Pathologists, Kelvin Grove 4059, Brisbane, QLD Australia; 3 School of Medicine, University of Queensland, Herston 4006, Brisbane, QLD Australia; 4 Department of Pathology, Colorectal Oncogenomics Group, Genetic Epidemiology Laboratory, The University of Melbourne, Parkville 3010, Melbourne, VIC Australia; 5 Division of Anatomical Pathology, SA Pathology, Adelaide, 5000 South Australia Australia; 6 Centre for Cancer Biology, University of South Australia, Adelaide, 5000 South Australia Australia; 7 Familial GI Cancer Service and Ministry of Health Bowel Cancer Programme, Auckland City Hospital, Auckland, New Zealand; 8 Centre for Epidemiology and Biostatistics, Melbourne School of Population and Global Health, The University of Melbourne, Parkville 3010, Melbourne, VIC Australia; 9 Department of Haematology and Oncology, The Queen Elizabeth Hospital, Woodville South 5011, Adelaide, South Australia Australia; 10 Flinders Medical Centre, Bedford Park 5042, Adelaide, South Australia Australia; 11 School of Medicine, University of Adelaide, Adelaide, 5000 South Australia Australia; 12 Cancer Epidemiology and Population Health, University of South Australia, Adelaide, 5000 South Australia Australia; 13 School of Nursing and Midwifery, Flinders University, Bedford Park 5042, Adelaide, South Australia Australia; 14 Basil Hetzel Institute, The Queen Elizabeth Hospital, Woodville South 5011, Adelaide, South Australia Australia; 15 Cancer Voices SA, Kensington Park 5068, Adelaide, South Australia Australia; 16 University of Adelaide Department of Surgery, The Queen Elizabeth Hospital, Woodville South 5011, Adelaide, South Australia Australia; 17 Cancer Theme, South Australian Health and Medical Research Institute, Adelaide, 5000 South Australia Australia; 18 Flinders Centre for Innovation in Cancer, Flinders University, Bedford Park 5042, Adelaide, South Australia Australia; 19 Bowel Health Service, Repatriation General Hospital, Daw Park 5041, Adelaide, South Australia Australia; 20 SAHMRI Colorectal Node, Basil Hetzel Institute, Woodville South, Adelaide, South Australia 5011 Australia

**Keywords:** Adenoma, Sessile serrated adenoma, Early-onset colorectal cancer

## Abstract

**Background:**

Colorectal cancer (CRC) diagnosed at <50 years is predominantly located in the distal colon and rectum. Little is known about which lesion subtypes may serve as CRC precursors in young adults. The aim of this work was to document the prevalence and histological subtype of lesions seen in patients aged <50 years, and any associated clinical features.

**Methods:**

An audit of the colonoscopy database at The Queen Elizabeth Hospital in Adelaide, South Australia over a 12-month period was undertaken. Findings were recorded from both colonoscopy reports and corresponding histological examination of excised lesions.

**Results:**

Data were extracted from colonoscopies in 2064 patients. Those aged <50 comprised 485 (24%) of the total. CRC precursor lesions (including sessile serrated adenoma/polyps (SSA/P), traditional serrated adenomas, tubular adenomas ≥10 mm or with high-grade dysplasia, and conventional adenomas with villous histology) were seen in 4.3% of patients aged <50 and 12.9% of patients aged ≥50 (*P* <0.001). Among colonoscopies yielding CRC precursor lesions in patients under 50 years, SSA/P occurred in 52% of procedures (11/21), compared with 27% (55/204) of procedures in patients aged 50 and older (*P* = 0.02). SSA/P were proximally located in (10/11) 90% of patients aged under 50, and 80% (43/54) of those aged 50 and older (*P* = 0.46).

**Conclusions:**

SSA/P were the most frequently observed CRC precursor lesions in patients aged <50. Most CRCs in this age group are known to arise in the distal colon and rectum suggesting that lesions other than SSA/P may serve as the precursor for the majority of early-onset CRC.

## Background

Colorectal cancer (CRC) in young adults (diagnosed before age 50 years) accounts for 7.5% of the total CRC burden in the Australian population [1], and its incidence has been increasing during the last two decades in both Australia and the USA [2–4]. Though the early age of onset for CRC is thought to reflect an increased genetic predisposition, the majority appear to be sporadic [5, 6]. CRC in young adults associated with known hereditary conditions such as Lynch syndrome comprises a minor component (15–17%) [7–10], whilst a further subset of cases have a clear familial basis with no attributable genetic aetiology [11]. Some authors have raised the possibility that sporadic early-onset CRC may result from the inheritance of multiple predisposing genetic variants, both rare and common [12], rather than the slow accumulation of somatic changes seen in sporadic CRC from older patients. Attempts to identify rare predisposing variants outside the known genetic syndromes have been undertaken on CRC patients under 40 years of age, and have encountered considerable heterogeneity, with few rare loss-of-function variants being recurrent [13]. Hence the origin of most early-onset CRC remains unexplained.

A number of authors have suggested that early-onset CRC is a distinct entity based on clinico-molecular analyses [14, 15]. Early-onset CRC occurs more frequently in the distal colorectum compared with CRC in older adults [3]. In addition, the CRC are more likely to display mucinous and signet ring cell differentiation, and high histologic grade. Early-onset CRC has also been reported to have a different molecular profile than that occurring in older adults [11]. In particular, early-onset CRC demonstrates less frequent *BRAF* mutation (7% vs 19%) and more frequent isolated *PIK3CA/PTEN* mutation (16% vs 1%) than tumours from patients aged over 70 at CRC diagnosis [16]. CRC develop from two *major* sub-classes of precursor lesions; conventional adenomas and serrated polyps. The molecular pathogenesis of CRC frequently reflects the lesion from which the CRC arises; however, studies of precursor lesion histological subtypes in the young are limited, as this age group is not undergoing population screening. Lesion subtypes are associated with a number of lifestyle risk factors [17, 18], and therefore may be studied to provide clues to the triggers for early-onset colorectal neoplasia. The aim of this study was to document the prevalence, location and histological subtype of CRC precursor lesions in patients undergoing colonoscopy before age 50 years in a hospital colonoscopy service and compare the findings with those from patients aged 50 years and older, with a view to increasing the current understanding of bowel neoplasia in young adults and seeking clues to its potential risk factors.

## Methods

This is a cross-sectional study using an audit of 2613 procedures recorded within the colonoscopy database at The Queen Elizabeth Hospital (a tertiary teaching hospital) in Adelaide, South Australia over a 12-month period from March 2013. Data from each procedure were extracted in sequence by a gastroenterology registrar (SW) and recorded in a purpose-dedicated database. Findings were obtained from both the colonoscopy report within a ProVation endoscopy procedure documentation system (Wolters Klewer) [19], and the corresponding pathology report concerning histological examination of excised lesions. Lesion sizes were derived from colonoscopy reports. Indications for colonoscopy were derived from admission notes in ProVation including self-reported family history of CRC (any non-specified relative with CRC), personal medical history of colonic neoplasia, bowel symptoms (pain, change of habit, diarrhoea, constipation), occult or overt bleeding, confirmed iron-deficiency anaemia, and confirmed or suspected inflammatory bowel disease. The study was approved by the Human Research Ethics Committee of the Central Adelaide Local Health Network under protocol number HREC/14/TQEHLMH/194.

### Definitions

Lesion subtypes were reported on a *per colonoscopy* basis. *Any serrated lesion* included hyperplastic polyps, sessile serrated adenoma/polyps (SSA/P) and traditional serrated adenomas (TSA) [20]. *Any conventional adenoma* comprised conventional adenomas with tubular and/or villous histology and low- or high-grade dysplasia. *CRC precursor lesions* (CPL), comprised SSA/P with or without dysplasia, TSA, tubular adenomas ≥10 mm in size or with high-grade dysplasia (ATA) and any conventional adenoma with villous histology (TVA or VA). *Proximal colon* refers to caecum, ascending colon, and transverse colon. *Distal colon* refers to splenic flexure, descending colon, sigmoid colon and rectum.

### Statistical analysis

Prevalence of characteristics of patients, indications for colonoscopy and findings were compared between two groups (<50 vs ≥50 years) using Pearson’s chi-squared test. Continuous variables were compared using a *t*-test. All statistical association tests were performed using SPSS Version 23 for Mac (IBM). Two-tailed statistics were used throughout with a significance level of *P*-value <0.05.

## Results

Of the total 2613 available reports, 398 (15%) were excluded due to incomplete data, and a further 77 procedures (3%) were not completed due to poor bowel preparation, looping or patient discomfort, leaving 2138 complete procedures (82%). Within this group, sixty-six patients underwent multiple colonoscopies during the study period. In instances of multiple procedures, data were drawn from the initial procedure only, leaving a total of 2064 individual patients whose data contributed to the study. Characteristics of patients and colonoscopy findings are given in Table 1.

**Table 1:** Characteristics of patients, and polyp findings in each age group

		All	%	Age <50	%	Age ≥50	%	*P*-value <50 vs ≥50
Totals		*n* = 2064		*n* = 485		*n* = 1579		
Mean age		59.7		39.2		66.0		
Median age		60		42		65		
Age range		18–96		18–49		50–96		
Sex	Males	984	47.6	220	45.4	764	48.4	0.13
	Females	1080	52.4	265	54.6	815	51.6	
Indications								
	IBD	90	4.4	49	10.1	41	2.6	*<0.001*
	Overt bleeding	348	16.8	108	22.2	240	15.2	*<0.001*
	Occult bleeding	350	16.9	29	5.9	321	20.3	*<0.001*
	Pain	227	11	77	15.9	150	9.5	*<0.001*
	Bowel habit	194	9.4	74	15.3	120	7.6	*<0.001*
	FH CRC	117	5.7	42	8.7	75	4.8	*0.001*
	PH CRC	83	4	4	0.8	79	5	*<0.001*
	PH polyps	303	14.9	34	7	274	17.4	*<0.001*
Findings								
	Any polyps	714	34.6	80	16.5	584	37	*<0.001*
	Serrated polyps	205	9.9	41	8.5	164	10.4	0.21
	Conventional adenomas	523	25.3	46	9.5	477	30.2	*<0.001*
	Multiple polyps	406	19.7	35	7.2	371	23.5	*<0.001*
	CPL	225	10.9	21	4.3	204	12.9	*<0.001*
Polyp sub-types								
	SSA/P	65	3.1	11	2.3	54	3.4	0.20
	TSA	1	0.01	0	0	1	0.06	
	TVA	95	4.6	8	1.6	87	5.5	*<0.001*
	ATA	76	3.7	3	0.6	73	4.6	*<0.001*
	VA	2	0.01	0	0	2	0.13	
Cancers								
	CRC	55	2.7	5	1	50	3.2	*0.01*
	Distal CRC Site	28	49.1	4	80	24	48.1	0.35

*IBD* Inflammatory bowel disease (Crohns disease, ulcerative colitis, NOS)*, FH CRC* Family history of CRC*, PH CRC* Personal history of CRC*, PH* Polyps Personal history of polyps*, CPL* known CRC precursor lesions*, SSA/P* Sessile serrated adenoma/polyps*, TVA* Tubulovillous adenoma*, ATA* Advanced tubular adenoma (≥10 mm, and/or high-grade dysplasia)*, TSA* Traditional serrated adenoma*, VA* Villous adenoma

Significant *p*-values are shown in italics

### Indications

Patients aged <50 were more likely to be having a procedure for *family history* of CRC (*P* = 0.001), whereas those aged ≥ 50 years were more likely to have a *personal history* of CRC or polyps (*P* <0.001). Five patients were known to have a DNA mismatch repair gene mutation (Lynch syndrome) prior to the procedure, and all were females and aged under 50 years. Confirmed or suspected inflammatory bowel disease, abdominal pain and change in bowel habit were more frequently reported as an indication for colonoscopy in young patients (*P* <0.001 in all instances). Young patients had a higher prevalence of overt bleeding as an indication for their procedure (*P* <0.001), whereas older patients demonstrated a higher prevalence of occult bleeding (*P* <0.001).

### Precursor polyps and cancers

**Fig. 1:**
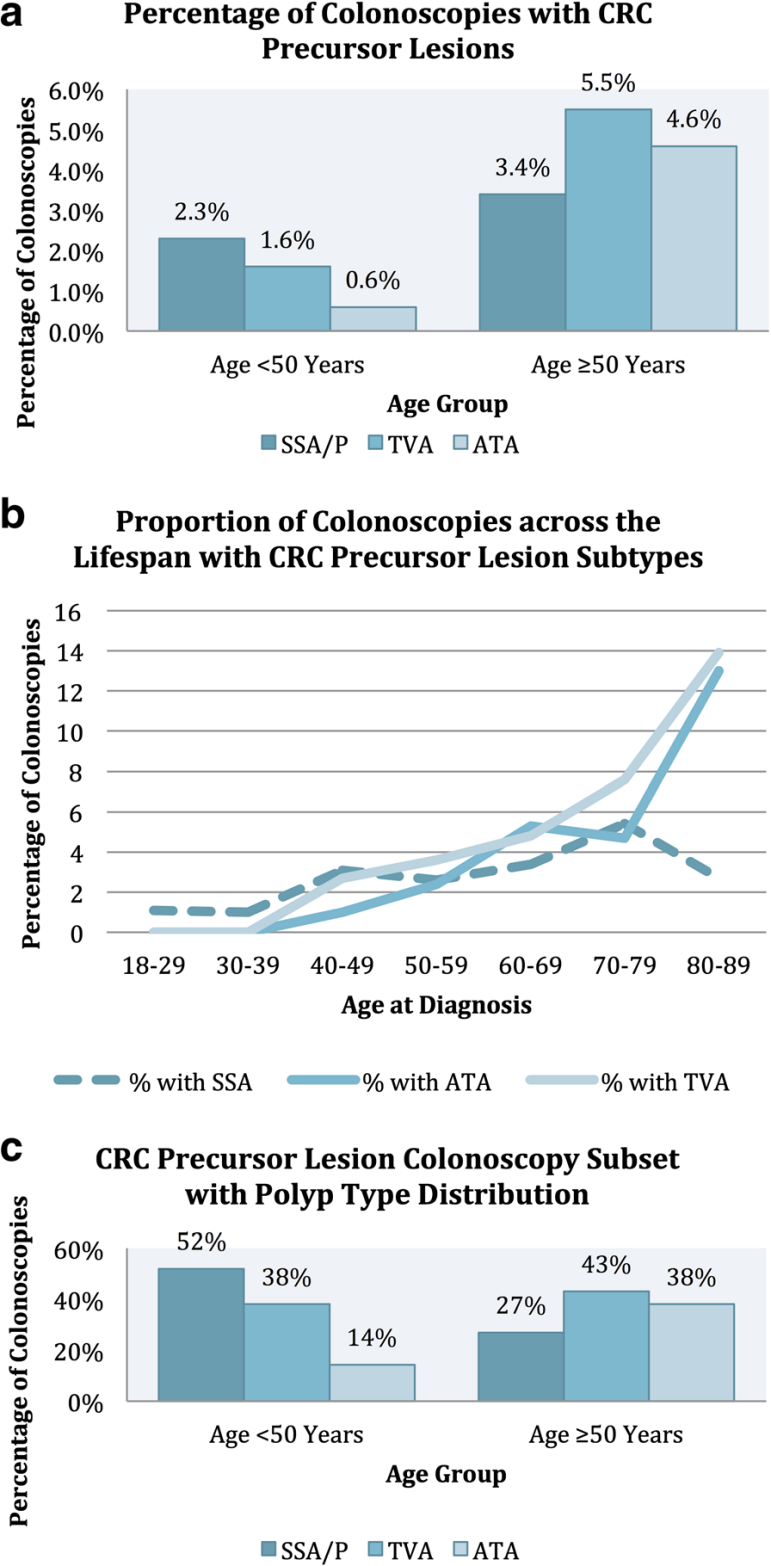
Distribution of Polyp Subtypes. Graphs showing the distribution of polyp subtypes **a** across all colonoscopies partitioned by age group, **b** within the colonoscopies which yielded known CRC precursor lesions (CPL) partitioned by age group and **c** the percentage of colonoscopies which yielded the three major subtypes of CRC precursor lesions, SSA/P, TVA and ATA across the lifespan. *SSA/P* sessile serrated polyp, *TVA* tubulovillous adenoma, *ATA* advanced tubular adenoma (≥10 mm or high-grade dysplasia)

Histologically confirmed neoplastic lesions were observed in 16.5% (80/485) of patients aged <50 and 37% (584/1579) of patients aged ≥50 (*P* <0.001) (Table 1). Polyps known to serve as CRC precursor lesions (CPL) were seen in 4.3% (21/485) of patients aged <50 and 12.9% (204/1579) of patients aged ≥50 (*P* <0.001). When all polyps were assessed according to subtype, there was no evidence for a difference in serrated polyp prevalence (any serrated subtypes including hyperplastic polyps) when data were partitioned at age 50 (*P* = 0.21). In contrast, conventional adenomas were more prevalent in the older age group (*P* <0.001). The distribution of CPL subtypes also differed between age groups (Table 1 and Fig. 1). The prevalence in the study population of SSA/P did not differ between the two age groups (*P* = 0.20) but both TVA (*P* <0.001) and ATA (*P* <0.001) were more prevalent in the older age group (Fig. 1a,b). SSA/P were removed from 11/21 (52%) colonoscopies bearing known precursor lesions in patients <50 year, compared with 55/204 (27%) colonoscopies in patients aged 50 and older (*P* = 0.02) (Fig. 1c). SSA/P were located in the proximal colon in 90% of cases within the patient group aged <50 years, and in 80% in those aged ≥50 (*P* = 0.45). Average size of SSA/P in the <50 age group was 6.8 mm (range 2–15 mm) and in those aged ≥50 was 8.7 mm (range 2–25 mm) (*P* = 0.36). There was no difference between the prevalence of SSA/P in each sex overall (*P* = 0.35) or within the younger patient age group (*P* = 0.59). The prevalence of CPL or CRC did not differ between the sexes in patients aged <50, however males were more likely than females to develop CPL or CRC in patients aged ≥50 years (*P* = 0.007) (Table 2). The service-based adenoma detection rate was 297/984 (30%) for males and 226/1080 (21%) for females.

**Table 2:** Medium to high-risk polyps or cancer grouped by sex and age

Group	Males	Percentage with CPL or cancer	Females	Percentage with CPL or cancer	*P*-value*
<50 years	11	5	15	5.7	0.75
**≥**50 years	140	18.3	109	13.4	*0.007*
All	151	15.3	124	11.5	*0.01*

*Comparison of proportion of each feature between males and females

*CPL* known CRC precursor lesions

Significant *p*-values are shown in italics

CRC was detected in 1.0% (5/485) of patients aged <50 vs 3.2% (50/1579) of patients aged ≥50 (*P* = 0.01). Colonoscopy-detected CRC in patients aged <50 was found in the distal colon in 4/5 (80%) compared with 24/50 (48%)(*P* = 0.35). Of the five patients known to have Lynch syndrome, only one was found to have CRC in the setting of long-standing ulcerative colitis, and the remaining four patients showed no pathology. All high-grade dysplasia in the audit was observed in the older age group in 16 polyps from 15 patients (12 females; average age 74). The polyps comprised 4 tubular adenomas and 11 tubulovillous adenomas. In addition, several rare polyp types were observed during the audit including a single, proximally located SSA/P with dysplasia in a 67 year old female, a villous adenoma in a 79 year old female, and a traditional serrated adenoma in a 51 year old female.

### Synchronously occurring lesion types

The co-occurrence of CPL subtypes in patients in each age group is shown in Table 3. Having at least one SSA/P was associated with any conventional adenoma across all patients (*P* = 0.002), with any conventional tubular adenoma (*P* = 0.001), whilst there was no evidence for an association with advanced tubular adenomas (*P* = 0.09) or tubulovillous adenomas (*P* = 0.99). The association between SSA/P and any conventional adenoma or any tubular adenoma remained significant when patients ≥50 years were considered (*P* = 0.02 and *P* = 0.008 respectively) but failed to attain statistical significance when patients aged under 50 years were analysed (*P* = 0.08 and *P* = 0.05 respectively). Co-occurrence between conventional adenomas, or tubular adenomas, and SSA/P was present regardless of family history of CRC.

**Table 3:** Co-occurrence of adenoma subtypes with SSA/P in patients with conventional adenomas

SSA/P co-occurring with…		SSA/P present (n)	(%)	SSA/P absent (n)	(%)	*P*-value*
Any AD	<50 years	3/11	27.3	43/474	9.1	0.08
	≥50 years	25/55	46.3	452/1523	29.7	*0.02*
	All	28/66	43.1	495/1997	24.8	*0.002*
Any TA	<50 years	3/11	27.3	37/474	7.8	0.05
	≥50 years	24/55	44.4	403/1524	26.4	*0.008*
	All	27/66	41.5	440/1998	22.0	*0.001*
ATA	<50 years	1/11	9.1	2/474	0.4	0.07
	≥50 years	4/55	7.4	69/1524	4.5	0.32
	All	5/66	7.7	71/1998	3.6	0.09
TVA	<50 years	0/11	0.0	8/485	1.7	0.99
	≥50 years	3/55	5.6	84/1524	5.5	0.99
	All	3/66	4.6	92/1998	4.6	0.99

*Comparison of proportions of specified condition present (i.e. SSA/P present among those with any adenoma, any tubular adenoma, an advanced tubular adenoma, or a tubulovillous adenoma) between young adults (<50 years) and older adults (≥50 years) compared to patients with the specified adenoma only

*SSA/P* sessile serrated adenoma/polyp, *Any AD* any conventional adenoma, *Any TA* any tubular adenoma, *ATA* advanced tubular adenoma (≥10 mm and/or high-grade dysplasia), *TVA* tubulovillous adenoma

Significant *p*-values are shown in italics

### Bleeding and polyp types

Patients who were having a colonoscopy for occult or active bleeding were more likely to have been diagnosed with CPL or CRC (*P* = 0.005) compared with patients not reporting any bleeding. This finding was seen in the older age group (*P* = 0.03), though not in younger patients (*P* = 0.18). When bleeding was stratified into overt and occult forms, occult bleeding was associated with CPL or CRC (*P* = 0.03), or with advanced conventional adenoma specifically (*P* = 0.009), in patients diagnosed at ≥50 years, but there was no evidence for this association in patients under 50 years. No associations emerged between bleeding and SSA/P in either age group (*P* = 0.99)(Table 4).

**Table 4:** Associations between significant neoplasia and bleeding observations in patients with bleeding

		Age <50	%	P-value	Age ≥50	%	*P*-value	All	%	*P*-value
		*n*=			*n*=			*n*=		
Totals	Patients with bleeding	137			561			698		
Any bleeding	With CPL/CRC	10	7.3	0.18	101	18.0	*0.03*	111	15.9	*0.005*
	With SSA/P	5	3.6	0.2	17	3.0	0.53	22	3.2	0.99
	With AA	4	2.9	0.54	65	11.6	*0.03*	69	9.9	*0.008*
Totals	Patients with overt bleeding	*n* = 108			*n* = 240			*n* = 348		
Overt bleeding	With CPL/CRC	8	7.4	0.17	40	16.7	0.31	48	13.8	0.34
	With SSA/P	4	3.7	0.21	8	3.3	0.56	12	3.4	0.42
	With AA	4	3.7	0.21	23	9.6	0.49	27	7.8	0.52
Totals	Patients with occult bleeding	*n* = 29			*n* = 321			*n* = 350		
Occult bleeding	With CPL/CRC	2	6.9	0.45	61	19.0	*0.03*	63	18.0	*0.002*
	With SSA/P	1	3.4	0.5	9	2.8	0.31	10	2.9	0.44
	With AA	0	0.0	0.5	42	13.1	*0.009*	42	12.0	*0.001*

*CPL/CRC* CRC precursor lesions and/or CRC, *SSA/P* sessile serrated polyp, *AA* advanced conventional adenoma (≥10 mm or high-grade dysplasia or villous histology)

Significant *p*-values are shown in italics

### Family history of CRC

There was no evidence for association between family history of CRC and CPL, or CRC specifically, in those aged under 50 at colonoscopy. There was some evidence for patients aged 50 and older to have an association between a family history of CRC and a diagnosis of CRC specifically (*P* = 0.08). SSA/P showed an association with family history of CRC (*P* = 0.04) in those aged 50 and older. No evidence for an association between family history of CRC and conventional adenomas was observed in either age group.

## Discussion

The results of this study suggest that, in contrast to older patients, SSA/P comprise a significantly higher *proportion* of the known CRC precursors [21] removed from patients aged under 50 in a routine colonoscopy service. Overall, SSA/P prevalence did not differ between the two age groups in the study, whereas advanced adenoma prevalence increased steadily with age, in parallel to some degree with the higher prevalence of cancer in the older age group, and consistent with what would be expected in the general population. A similar pattern to these observations has been reported previously among all polyp subtypes [22]. In a retrospective cohort of 28,544 asymptomatic patients from Korea aged 20-88 years, and undergoing a work-related health check, the prevalence of serrated lesions was seen to be higher than that of conventional adenomas in youngest age groups, and to increase *only slightly* with age. Conventional adenomas, in contrast, had a lower prevalence than serrated polyps in the youngest age group and increased in prevalence with increasing age. The authors of the Korean study concluded that serrated polyp prevalence, including that of SSA/P, were relatively high in patients aged under 50 years. Though extensive studies have been reported from Asian cohorts which document colorectal neoplasia in patients aged under 50 years [22–24], this current study reports the findings at colonoscopy in young adults from a predominantly Caucasian population. The prevalence of hyperplastic (serrated) polyps per se observed in this study is comparable with that reported by Wong et al [23], however a recent report from Australia suggests that SSA/P subtype is relatively rare in Chinese patients when compared with Caucasians [25].

In both age groups, approximately 80% of SSA/P arose in the proximal colon. In patients under 50 with CRC, the majority of malignant lesions was observed in the distal colon and rectum in this report, and this is consistent with the current understanding that early-onset CRC is predominantly a malignancy of the distal large bowel [3, 12, 26, 27]. SSA/P, being located in the proximal colon in young adults, are therefore unlikely to be the precursor lesion for most early-onset CRC. In support of this premise, somatic *BRAF* mutation, the molecular genetic hallmark of SSA/P, is relatively rare in CRC from young adults, being present at (3/45) 7% in a Norwegian study [16], 1/68 (2%) in a Spanish report [8], and in 0/39 (0%) from France [14]. Activating mutations in *BRAF* are present in 14–16% of all population-based CRC [28, 29].

It is not known for certain whether SSA/P have always been present in young patients undergoing colonoscopy. Indeed, growing awareness of SSA/P has occurred only in recent decades, and may have contributed to this observation. Alternatively, the presence of SSA/P in young patients may be a surrogate marker for mucosal abnormalities and future risk of advanced neoplasia, related to an emerging population risk factor among young adults. Smoking has been consistently associated with SSA/P, and, though it is thought by some authors that the induction time in young adults is insufficient to produce neoplasia [3, 30, 31], a recent report has presented evidence that it may be a contributory factor [32]. Associations between obesity and diabetes and a diagnosis of SSA/P have been reported inconsistently [33, 34]. However, metabolic risk scores for prediction of advanced colorectal neoplasia demonstrate their greatest effects in patients aged under 60 years [35]. More recently, diabetes has been identified as an independent risk factor for colorectal neoplasia in patients aged 40–49 years [23] and diabetes has been identified elsewhere as a risk factor for early-onset CRC [36].

The study also incidentally highlights a number of features of SSA/P. It presents indirect evidence for a prolonged dwell time for these lesions, and suggests that SSA/P may arise early and be present in the colon for decades without a significant increase in size [37]. Bleeding as an indication for colonoscopy was not associated with SSA/P in our data [38], in contrast to this symptom showing a significant association with advanced conventional adenomas. In addition, the study confirms a previous observation of an association between SSA/P and a family history of CRC [39], though this was not apparent in patients under 50. In patients under 50, the lower overall age of first-degree relatives may be a contributing factor to this observation.

The study observed a significant likelihood for SSA/P to co-occur with *non-villous* conventional adenomas specifically, though association between SSA/P and conventional adenomas generally has long been recognised [38, 40–44]. A recent report provided indirect evidence for our finding in that conventional adenomas which occur synchronously with SSA/P lack *KRAS* mutation [45] which is a known feature of villous histology [46]. A further report has shown that proximal serrated polyps have been associated with multiple non-advanced conventional adenomas in Chinese patients [43]. These findings may be related to a predisposed mucosa [47], and as such may serve as a *marker* for increased risk of advanced neoplasia, rather than as precursors per se. The presence of small SSA/P in association with a small tubular adenoma has recently been associated with a significantly increased rate of metachronous advanced neoplasia during surveillance when compared to small tubular adenomas alone (18.2% vs 7.8%, *P* = 0.02). In addition, the presence of small SSA/P had a risk of metachonous advanced neoplasia comparable with an index high-risk conventional adenoma (17.9% for a small isolated SSA/P vs 15.9% for a high-risk conventional adenoma) [48]. It has been suggested that isolated small SSA/P therefore be considered for new surveillance interval recommendations [48]. These observations highlight the clinical relevance of findings in our current study, as there may be potential benefit of increased longitudinal follow-up in young adults with SSA/P, *regardless of size*. That there was no association between SSA/P and adenomas with villous histology may reflect genetic background in patients who develop SSA/P [49].

A previous report has suggested that rectal bleeding is less useful for prediction of advanced neoplasia in patients under 50 years [50], and this observation has also emerged in our study. The differences in occult versus active bleeding may have some implications when we look at mechanisms to reduce the impact from an increasing rate of CRC in patients aged under 50 years. Only 6% of those <50 years had occult bleeding compared to 20.3% for those aged over 50 years (*p* = <0.001). The low rate may reflect a lower uptake of the testing, as expected, because young patients are not undergoing screening. It also highlights a difference in timing of presentation with younger patients more likely to await symptoms and there is evidence of more advanced disease potentially supportive of this [51]. Although education around testing for occult bleeding will still be relevant, more general education approaches around screening, symptoms and early presentation to a health care provider may be just as important, both targeting patients and the health care profession, and may involve other markers of increased risk such as family history of malignancy and personal history of diabetes.

The study has a number of limitations. Patients under 50 are undergoing procedures for a different range of indications, although this does reflect real world practice. The number of young adults with neoplastic findings was relatively low and may have affected the power of a subset of comparisons. However, the presence of SSA/P in young adults is an important observation which has the potential to identify those aged under 50 who are at higher than population risk. In addition, the population did not include cases of advanced neoplasia diagnosed by sigmoidoscopy or imaging, and it is difficult to know whether this has influenced the ability to generalise to all young adults undergoing investigations. Colonoscopies were performed at a single tertiary centre involving multiple colonoscopists, and individual adenoma detection rates were not available. However, our service-based adenoma detection rate is within the updated limits suggested for a quality colonoscopy service of at least 30% for males and 20% for females [52]. Family history was self-reported and not systematically verified, and indications for colonoscopy were extracted from admission notes. Allowing for these limitations, results showed that adenoma prevalence rises with age, and this is in keeping with what would be expected in the general population [53]. This report also confirms a recent publication demonstrating that advanced polyps in patients under 45 are more likely to be sporadic than associated with family history, as family history of CRC was reported in 7.1% of those with any advanced neoplasia [54].

## Conclusions

In summary, proximal SSA/P comprised a greater proportion of lesions with malignant potential in patients under age 50, compared with older patients. However most CRC in this age group occurs in the distal colon and rectum, both in this study, and consistently in the literature [3, 12, 26, 27], suggesting that SSA/P are unlikely to serve as the premalignant lesion for the majority of early-onset CRC, but may have a potential role as *markers* of future increased risk for advanced neoplasia. Further, SSA/P in both age groups co-occurred significantly with conventional non-villous adenomas, a combination with increased risk for subsequent advanced neoplasia [48].

## Abbreviations

AA: Advanced conventional adenoma
ATA: Advanced tubular adenoma (≥10 mm, and/or high-grade dysplasia)
CPL: Known CRC precursor lesions
FH CRC: Family history of CRC
IBD: Inflammatory bowel disease (Crohns disease, ulcerative colitis, NOS)
PH CRC: Personal history of CRC
PH Polyps: Personal history of polyps
SSA/P: Sessile serrated adenoma/polyp
TSA: Traditional serrated adenoma
TVA: Tubulovillous adenoma
VA: Villous adenoma
